# Disease progression and treatment response in data-driven subgroups of type 2 diabetes compared with models based on simple clinical features: an analysis using clinical trial data

**DOI:** 10.1016/S2213-8587(19)30087-7

**Published:** 2019-06

**Authors:** John M Dennis, Beverley M Shields, William E Henley, Angus G Jones, Andrew T Hattersley

**Affiliations:** a Institute of Biomedical and Clinical Science, Royal Devon and Exeter Hospital, University of Exeter Medical School, Exeter, UK; b Health Statistics Group, Institute of Health Research, Royal Devon and Exeter Hospital, University of Exeter Medical School, Exeter, UK

## Abstract

**Background:**

Research using data-driven cluster analysis has proposed five subgroups of diabetes with differences in diabetes progression and risk of complications. We aimed to compare the clinical utility of this subgroup-based approach for predicting patient outcomes with an alternative strategy of developing models for each outcome using simple patient characteristics.

**Methods:**

We identified five clusters in the ADOPT trial (n=4351) using the same data-driven cluster analysis as reported by Ahlqvist and colleagues. Differences between clusters in glycaemic and renal progression were investigated and contrasted with stratification using simple continuous clinical features (age at diagnosis for glycaemic progression and baseline renal function for renal progression). We compared the effectiveness of a strategy of selecting glucose-lowering therapy using clusters with one combining simple clinical features (sex, BMI, age at diagnosis, baseline HbA_1c_) in an independent trial cohort (RECORD [n=4447]).

**Findings:**

Clusters identified in trial data were similar to those described in the original study by Ahlqvist and colleagues. Clusters showed differences in glycaemic progression, but a model using age at diagnosis alone explained a similar amount of variation in progression. We found differences in incidence of chronic kidney disease between clusters; however, estimated glomerular filtration rate at baseline was a better predictor of time to chronic kidney disease. Clusters differed in glycaemic response, with a particular benefit for thiazolidinediones in patients in the severe insulin-resistant diabetes cluster and for sulfonylureas in patients in the mild age-related diabetes cluster. However, simple clinical features outperformed clusters to select therapy for individual patients.

**Interpretation:**

The proposed data-driven clusters differ in diabetes progression and treatment response, but models that are based on simple continuous clinical features are more useful to stratify patients. This finding suggests that precision medicine in type 2 diabetes is likely to have most clinical utility if it is based on an approach of using specific phenotypic measures to predict specific outcomes, rather than assigning patients to subgroups.

**Funding:**

UK Medical Research Council.

## Introduction

Type 2 diabetes is a heterogeneous, multifactorial condition, comprising 90–95% of all cases of diabetes and affecting over 400 million people worldwide. There is great interest in better characterising the heterogeneity in type 2 diabetes and in exploiting this heterogeneity to improve care and outcomes for individuals with type 2 diabetes.^1^, ^2^, ^3^

Ahlqvist and colleagues^4^ identified five replicable clusters of individuals with diabetes in the All New Diabetics in Scania (ANDIS) cohort. The smallest cluster was defined by the presence of glutamic acid decarboxylase autoantibody (GADA), regardless of other characteristics (cluster 1: severe autoimmune diabetes [SAID]). Four type 2-like clusters were then characterised by the absence of GADA positivity and varying degrees of differences in age at diagnosis, and baseline measures of BMI, HbA_1c_, and homoeostatic model assessment (HOMA) 2 measured insulin resistance and β-cell function. The four type 2 diabetes clusters were cluster 2, severe insulin-deficient diabetes (SIDD); cluster 3, severe insulin-resistant diabetes (SIRD); cluster 4, mild obesity-related diabetes (MOD); and cluster 5, mild age-related diabetes (MARD). Ahlqvist and colleagues showed potentially clinically important differences in disease progression and risk of complications between the clusters in observational follow-up, most notably a striking increase in the risk of diabetic kidney disease in cluster 3 (SIRD).

The key question for any subgroup analysis is the clinical utility of the subgroups, and in particular whether the proposed subgroups differ in response to therapy, which could help to inform treatment strategies.^2^ Ahlqvist and colleagues suggested but did not show that the clusters could be useful to guide choice of therapy.^5^ The only stratified approaches in type 2 diabetes showing large differences in response between treatments have used subgroups defined by routine clinical measures such as sex and BMI.^6^ A further key question, raised by van Smeden and colleagues^7^ in response to the original study, is whether assigning individuals to clusters has greater clinical utility for predicting outcomes than an approach that combines continuous clinical features to predict outcomes for individual patients.

**Research in context**

**Evidence before this study**

A study by Ahlqvist and colleagues proposed a novel stratification method for patients with diabetes, using a data-driven cluster analysis in Scandinavian registry data to identify five reproducible subgroups of adult-onset diabetes. The authors showed differences between the clusters in disease progression and risk of complications in observational follow-up. The authors suggested the clusters might help with therapy selection in the future but did not test whether the clusters could inform therapy choice. We searched Scopus, Web of Science, and Google Scholar for citations of the original study, searching for follow-up studies assessing the reproducibility, clinical utility, and role in treatment selection of the proposed data-driven clusters up to Jan 1, 2019. We identified a study that identified similar clusters in a Chinese population and a small mixed American population but that did not examine any aspect of clinical utility because clinical follow-up was not available. A second study of Danish patients applied a similar cluster analysis and, with duration of diabetes as an additional input variable, identified five subgroups of type 2 diabetes that differed to those in the original study, and differed in the prevalence of diabetes complications. No studies were found that tested the clinical utility and the role in treatment of the proposed cluster-based approach.

**Added value of this study**

This study advances the concept of heterogeneity in type 2 diabetes by testing the clinical utility of the data-driven cluster approach proposed by Ahlqvist and colleagues. The cluster analysis was repeated, and differences by cluster in disease progression and treatment response were assessed in newly diagnosed participants in the ADOPT trial with randomised, protocol-driven, follow-up data available. We found that the clusters were reproducible and differed in progression and treatment response. However, simple clinical measures were as or more useful than were the clusters for stratifying each outcome assessed.

**Implications of all the available evidence**

Patients with type 2 diabetes differ in treatment response and risk of disease progression, raising the possibility of a practical, stratified approach that is clinically orientated. Our study suggests a prediction model approach, combining phenotypic measures to predict specific outcomes for individual patients, is likely to have greater clinical utility than approaches that use clinical features to assign individuals to subgroups.

We aimed to establish the clinical utility of the clusters by analysing two large existing trial datasets of individuals randomised to metformin, sulfonylurea, and thiazolidinedione therapy, ADOPT and RECORD.^8^, ^9^ By contrast with the observational follow-up by Ahlqvist and colleagues,^4^ these trial datasets provided protocol-driven, randomised follow-up to assess clinical outcomes and differences in response to therapy. We compared the utility of the data-driven clusters with simpler approaches based on routine clinical measures available in any diabetes clinic.

## Methods

### Study population

The primary study population comprised newly diagnosed, drug-naive, individuals with type 2 diabetes, recruited between April, 2000, and June, 2002, followed up until June, 2006, who had participated in the ADOPT trial of glycaemic durability, randomly assigned to metformin, sulfonylurea (glibenclamide), or thiazolidinedione (rosiglitazone) monotherapy for up to 5 years (n=4351).^8^ Eligibility criteria at screening included age 30–75 years, fasting plasma glucose 7–13 mmol/L, and no evidence of renal impairment (serum creatinine >114 μmol/L for men or >106 μmol/L for women). As a replication dataset we used participants in the RECORD study^9^ (n=4447), a cardiovascular outcomes trial in individuals with established type 2 diabetes (mean duration of diabetes 7·1 [SD 4·9] years), initiating the same drug classes as in ADOPT but as dual second-line therapy, for up to 6 years. Sulfonylurea type was chosen on the basis of local practice (glibenclamide [18%], gliclazide [30%], or glimepiride [52%]) and rosiglitazone was the thiazolidinedione used. Eligibility criteria included age 40–75 years, BMI greater than 25·0 kg/m^2^, HbA_1c_ 7·0–9·0% (53–75 mmol/mol), and no evidence of renal impairment (serum creatinine >130 μmol/L) for men and women.

We followed up individuals from ADOPT and RECORD from randomisation until the earliest of: the primary outcome of the original trial, censor date, 5 years, or the occurrence of an outcome of interest. Full individual-level trial data were accessed through Clinical Trial Data Transparency Portal (Proposal 930).

### Procedures

In ADOPT we calculated HOMA2 measures of insulin resistance and β-cell function with fasting C-peptide and fasting-glucose measures using the HOMA2 calculator.^10^ GADA positivity (yes or no) was measured using a commercially available radioimmunoassay.^11^ In RECORD, we calculated HOMA2 measures using fasting insulin because data on fasting C-peptide were not available. GADA was not measured. Sex, age at diagnosis, baseline BMI, and baseline HbA_1c_ comprised the other measures required for cluster analysis.

### Outcomes

Glycaemic progression was defined as the change in HbA_1c_ from 1 year up to 5 years, thus allowing for an initial period of treatment response up to 1 year.

Chronic kidney disease was defined as progression from normal glomerular filtration rate (estimated glomerular filtration rate [eGFR] ≥60 mL/min per 1·73 m^2^) to confirmed chronic kidney disease stage 3 (two consecutive measures of eGFR <60 mL/min per 1·73 m^2^). eGFR was calculated using the chronic kidney disease epidemiology collaboration (CKD-EPI) equation;^12^ as a sensitivity analysis eGFR was calculated using the Modification of Diet in Renal Disease (MDRD) Study equation.^13^ Measures of renal function were recorded at baseline, 6 months, and annually. If progression was confirmed, the first of the two study visits was used to define chronic kidney disease onset. Albuminuria was defined as progression from normal urinary albumin to creatinine ratio (UACR, <30 mg/g) to either microalbuminuria (UACR 30–300 mg/g) or macroalbuminuria (UACR ≥300 mg/g). Individuals with eGFR lower than 60 mL/min per 1·73 m^2^ were excluded from the analysis of chronic kidney disease and those with UACR 30 mg/g or more at their baseline visit were excluded from analysis of albuminuria outcomes.

Glycaemic response was defined as achieved HbA_1c_ and as cumulative HbA_1c_ reduction at 3 years, as measured by area-under-the-curve (3-year AUC HbA_1c_). AUC HbA_1c_ is equivalent to the time-updated HbA_1c_ measure used in the UK Prospective Diabetes Study outcomes model.^14^ 3 years was chosen as the timepoint at which average AUC HbA_1c_ was approximately equal between the three drugs.^8^ Other timepoints will tend to favour a specific therapy; early timepoints will favour sulfonylureas because these drugs have an increased short-term response, but later timepoints favour thiazolidinediones, which have increased glycaemic durability.^8^

### Cluster analysis

In ADOPT, we repeated the clustering approach of Ahlqvist and colleagues.^4^ Men and women were clustered separately then pooled, continuous measures were mean centred and standardised, and continuous measures greater than five SDs from the mean were excluded. We applied K-means clustering specifying four clusters to the GADA-negative subset of individuals because K-means clustering does not incorporate binary variables; all GADA-positive individuals were manually assigned to a separate cluster.^4^ The same R command (kmeansrun), number of runs (100), and measure of cluster stability (Jaccard coefficient >0·75, after 2000 bootstraps) were applied.^15^ Once clusters were defined we assigned the same cluster names as in the original study, based on the distribution of cluster characteristics. In RECORD, we first assigned each individual to their ADOPT-derived cluster on the basis of their Euclidean distance from each cluster centre, and second, repeated the cluster analysis to derive RECORD-specific clusters. As GADA status was not available, all individuals in RECORD were assumed to be GADA-negative.

### Statistical analysis

In both cohorts, mean HbA_1c_ trajectories from randomisation up to 5 years for each cluster were first estimated using a repeated-measures mixed-effects model, including fixed effects for study visit, assigned cluster, and a study visit by cluster interaction. Patient-level random effects and an unstructured covariance matrix were specified for this and subsequent mixed-effects models. All individuals within a trial were pooled to assess progression, regardless of randomised therapy. To estimate glycaemic progression by cluster, the same model was then fitted but with HbA_1c_ change from 1 year as the outcome. We estimated the mean annual rate of glycaemic progression for each cluster by updating the cluster model to replace study visit with time as a linear covariate. Mean HbA_1c_ by age was estimated using the same model but with a linear term for continuous age at diagnosis replacing the clusters. For each model we estimated the proportion of variance explained (R^2^) by the fixed effects, Akaike information criterion, and the adequacy index.^16^, ^17^

We compared the cumulative incidence of chronic kidney disease by cluster using Kaplan-Meier plots and both unadjusted and baseline eGFR (continuous linear term) adjusted Cox proportional hazard models with cluster as a categorical variable. We estimated R^2^ and the discrimination ability (Harrell's C-index) of the unadjusted cluster Cox model compared with a Cox model with continuous baseline eGFR as a linear term.^17^ We repeated the same analysis for time to a 30% decline in eGFR, and for time to albuminuria with and without adjustment for baseline UACR as a continuous linear term. We also compared continuous relative changes from baseline in eGFR and UACR progression by cluster, using mixed-effects models with fixed effects for study visit, cluster, and study visit by cluster interaction.

We tested whether HbA_1c_ response to the three drugs differed across the clusters in ADOPT. Average HbA_1c_ trajectories by drug were estimated up to 3 years for each cluster separately, using repeated-measures mixed-effects models with fixed effects for study visit, drug, visit by drug interaction, and visit by baseline HbA_1c_ interaction. 3-year AUC HbA_1c_ was estimated for each drug in each cluster as the integral of the area under the mean HbA_1c_ trajectory, using the trapezoidal rule.

### Treatment selection: clusters strategy *vs* clinical features strategy

We investigated whether clusters were more useful than simple clinical features to select a drug for individual patients based on predicted 3-year AUC HbA_1c_. Models to predict HbA_1c_ were developed in ADOPT using two strategies: (1) using the clusters and (2) using clinical features. For the clusters strategy, we estimated HbA_1c_ response for each drug at the cluster level and applied this to all individuals within the cluster. This strategy treats individuals within a cluster as homogenous for treatment response to a particular drug. For the clinical features strategy, we combined sex and linear terms for age at diagnosis, baseline BMI, and baseline HbA_1c_ (the four routine clinical features informing the clusters) in a multivariable model to estimate HbA_1c_ response specific to each individual for each drug. The benefit of using each strategy developed in the ADOPT trial to select treatment for individuals was then tested in an external trial population, RECORD.

For the clusters strategy, 3-year AUC HbA_1c_ for each drug was estimated at cluster level. For the clinical features strategy, 3-year AUC HbA_1c_ was estimated for each individual on the basis of their precise clinical characteristics, using multivariable repeated-measures mixed-effects models for each drug. Each model had HbA_1c_ up to 3 years as the outcome, with age at diagnosis, BMI, baseline HbA_1c_, and study visit by baseline HbA_1c_ interaction as continuous linear terms, and study visit and sex as fixed effects. Model performance for each strategy was assessed using R^2^.

The purpose of a treatment selection model is to select the most effective therapies for individual patients, and therefore improve outcome at a population level, rather than to predict drug response accurately. Therefore, the true test of a treatment selection model is whether it can robustly identify individuals who are likely to benefit from particular therapies.^18^ Standard model performance metrics test the ability of a model to predict the outcome and are therefore insufficient in this context.^18^, ^19^

We therefore applied the following steps to test the effectiveness of each treatment selection strategy. For each individual in RECORD, we applied the models developed in ADOPT to obtain estimates of 3-year AUC HbA_1c_ on each drug. In the clusters strategy, these predictions were according to the individual's assigned cluster (the same for all individuals within a cluster). In the clinical features strategy, predictions were made at the individual level, estimated from precise clinical features. For each strategy, we applied a simple decision rule to assign individuals into two groups, one concordant and one discordant. Discordant individuals were those randomly assigned to a drug with a predicted 3-year AUC HbA_1c_ that is 3 mmol/mol higher (ie, less improvement in HbA_1c_) than that of the drug predicted to be their best drug; all other individuals were defined as concordant.^20^ The effectiveness of each treatment selection strategy was measured by the difference in 3-year AUC HbA_1c_ between the concordant and discordant groups. 3-year AUC HbA_1c_ by concordant or discordant group was estimated from a mixed-effects model with study visit, concordant or discordant group, baseline HbA_1c_, study visit by concordant or discordant group interaction, and visit by baseline HbA_1c_ interaction as fixed effects. We tested the sensitivity of results to the HbA_1c_ threshold used to define concordance by repeating the analysis at HbA_1c_ thresholds of 0, 1, 2, and 4 mmol/mol.

In RECORD, we compared the time to the trial primary outcome, cardiovascular hospitalisation or cardiovascular death, by cluster using unadjusted and baseline age-adjusted Cox proportional hazard models.

To examine the utility of the original ANDIS clusters, we assigned individuals in ADOPT to their ANDIS cluster on the basis of their Euclidean distance from the cluster centres defined by Ahlqvist and colleagues.^4^ We estimated glycaemic and renal progression and HbA_1c_ response for each ANDIS-derived cluster and compared model performance of the ADOPT defined clusters and ANDIS clusters. All analyses were done using R (version 3.4.1).

### Role of the funding source

The funders had no role in the study design, data collection, data analysis, data interpretation, or writing of the report. The corresponding author had full access to all data and had final responsibility for the decision to submit for publication.

## Results

**Figure 1:**
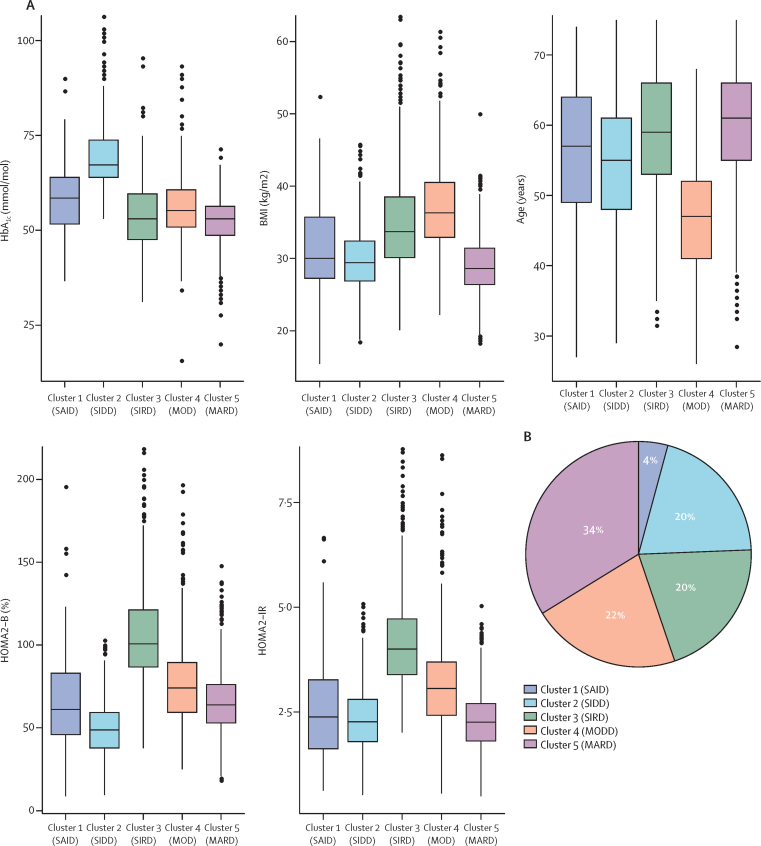
Cluster characteristics and cluster distribution in ADOPT (A) Distributions of HbA_1c_, BMI, age at diagnosis, HOMA2-B, and HOMA2-IR at baseline for each cluster. (B) Distribution of ADOPT participants (n=4003) according to k-means clustering. SAID=severe autoimmune diabetes. SIDD=severe insulin-deficient diabetes. SIRD=severe insulin-resistant diabetes. MOD=mild obesity-related diabetes. MARD=mild age-related diabetes. HOMA2-B=homoeostatic model assessment 2 estimates of β-cell function. HOMA2-IR=homoeostatic model assessment 2 estimates of insulin resistance.

We found that the clusters identified by Ahlqvist and colleagues were reproducible in trial populations. 4003 individuals in ADOPT had valid baseline measures for cluster assignment. Of these, 3802 were in the intention-to-treat population and so were eligible for analysis of patient outcomes. We found a clear pattern of differences between clusters in clinical characteristics (figure 1A, appendix pp 3–5), and were able to assign the same cluster names as Ahlqvist and colleagues did (figure 1B). Clusters were reasonably stable (Jaccard mean range for men 0·76–0.82; for women 0·69–0·82). Cluster-centre coordinates are shown in the appendix (p 3). In RECORD, 4148 individuals were eligible for cluster assignment (4057 in the intention-to-treat population). RECORD clusters were similar to the ADOPT clusters whether they were assigned from ADOPT or defined de novo in RECORD (appendix p 6).

**Figure 2:**
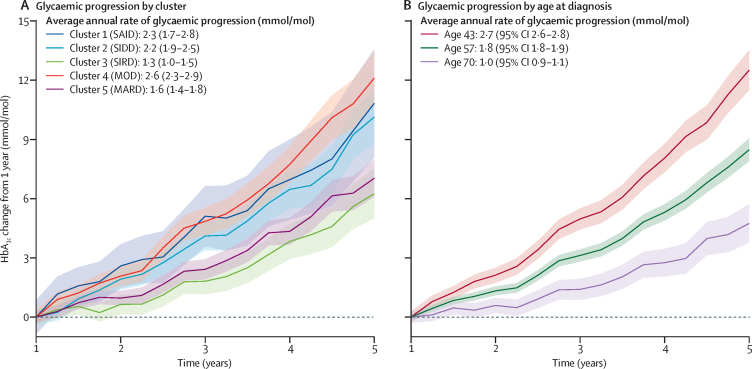
Glycaemic progression by cluster in ADOPT from 1 to 5 years (A) HbA_1c_ change by cluster (n=3016). (B) HbA_1c_ change by age at diagnosis (10th, 50th, and 90th percentile of ADOPT participants. Data are estimates from repeated measures, mixed-effects models. SAID=severe autoimmune diabetes. SIDD=severe insulin-deficient diabetes. SIRD=severe insulin-resistant diabetes. MOD=mild obesity-related diabetes. MARD=mild age-related diabetes.

Average HbA_1c_ trajectories by cluster from randomisation to 5 years are shown in the appendix (p 7). Glycaemic progression from 1 year differed by cluster in ADOPT (figure 2A), with a higher rate of progression in clusters 1 (SAID), 2 (SIDD), and 4 (MOD). In RECORD, only cluster 4 (MOD) had a higher rate of progression (appendix p 8). However, in both trials older age at diagnosis was associated with a lower rate of glycaemic progression (mean annual difference in rate of HbA_1c_ change per year increase in age at diagnosis: (ADOPT −0·06 mmol/mol, 95% CI −0·07 to −0·05; RECORD −0·05 mmol/mol, 95% CI −0·06 to −0.04; figure 2B, appendix p 8). Age at diagnosis explained a similar proportion of variation in progression to the clusters (ADOPT R^2^=0·09 age at diagnosis, R^2^=0·08 clusters; RECORD R^2^=0·05 age at diagnosis, R^2^=0·05 clusters). Other measures of model performance were also similar (appendix p 8).

**Figure 3:**
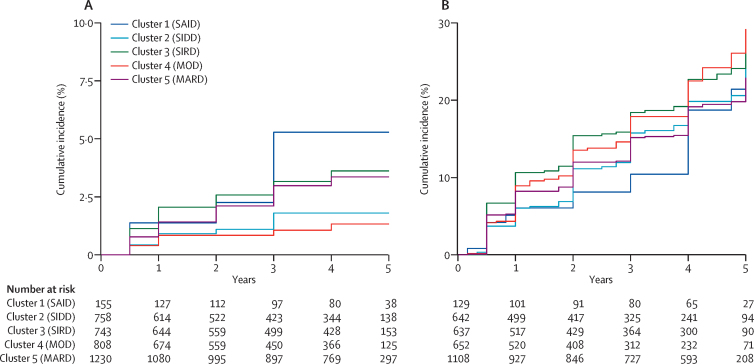
Renal progression by cluster in ADOPT over 5 years (A) Cumulative incidence of chronic kidney disease stage 3 (confirmed eGFR <60 mL/min per 1·73 m^2^) in individuals with eGFR ≥60 mL/min per 1·73 m^2^ at baseline (n=3694). (B) Cumulative incidence of albuminuria (UACR ≥30 mg/g) in individuals with UACR <30 mg/g at baseline (n=3168). eGFR=estimated glomerular filtration rate. UACR=urinary albumin to creatinine ratio. SAID=severe autoimmune diabetes. SIDD=severe insulin-deficient diabetes. SIRD=severe insulin-resistant diabetes. MOD=mild obesity-related diabetes. MARD=mild age-related diabetes.

We found differences in the incidence of chronic kidney disease between clusters after excluding patients with pre-existing chronic kidney disease; clusters 1, 3, and 5 had the highest incidence of chronic kidney disease (figure 3A, appendix p 9). However, there were differences between the clusters in baseline renal function: the clusters with the highest incidence of chronic kidney disease had the lowest baseline eGFR (appendix p 5). After adjustment for baseline eGFR there was no evidence of a difference in time to chronic kidney disease across the clusters (table 1, appendix p 10). Results were similar using eGFR calculated using MDRD (appendix p 11). In ADOPT, baseline eGFR explained a greater proportion of variation (R^2^=0·18) and discrimination ability (C-statistic 0·90) than did the clusters (R^2^=0·01, C-statistic 0·58); these results were similar to those in RECORD (baseline eGFR R^2^=0·15, C-statistic 0·86; clusters R^2^=0·01, C-statistic 0·57). Relative change from baseline in eGFR and time to 30% decline in eGFR did not differ by cluster (appendix pp 12–15).

**Table 1:** Risk of renal progression by cluster in ADOPT

	**Participants**	**Person-years at risk**	**Events**	**Unadjusted HR (95% CI)**	**HR (95% CI)**
**Time to CKD stage 3 (n=3694)**					
Cluster 1 (SAID)	155	499	6	2·82 (1·02−7·75)	1·56 (0·56−4·29)*
Cluster 2 (SIDD)	758	2262	10	1·00 (ref)	1·00 (ref)*
Cluster 3 (SIRD)	743	2501	22	2·05 (0·97–4·33)	1·11 (0·53–2·35)*
Cluster 4 (MOD)	808	2428	8	0·74 (0·29–1·88)	1·43 (0·56–3·63)*
Cluster 5 (MARD)	1230	4369	34	1·84 (0·91–3·73)	1·39 (0·69–2·82)*
**Time to albuminuria (n=3168)**					
Cluster 1 (SAID)	129	381	20	0·96 (0·59–1·55)	1·24 (0·76–1·52)†
Cluster 2 (SIDD)	642	1669	93	1·00 (ref)	1·00 (ref)†
Cluster 3 (SIRD)	637	1781	121	1·23 (0·94–1·62)	1·32 (1·01–1·73)†
Cluster 4 (MOD)	652	1630	108	1·19 (0·90–1·56)	1·27 (0·96–1·67)†
Cluster 5 (MARD)	1108	3428	183	0·98 (0·76–1·26)	1·18 (0·92–1·52)†

* Adjusted for baseline eGFR.

† Adjusted for baseline UACR. HR=hazard ratio. eGFR=estimated glomerular filtration rate. CKD=chronic kidney disease. SAID=severe autoimmune diabetes. SIDD=severe insulin-deficient diabetes. SIRD=severe insulin-resistant diabetes. MOD=mild obesity-related diabetes. MARD=mild age-related diabetes. UACR=urinary albumin to creatinine ratio.

There was no clear pattern of difference between clusters in baseline UACR (appendix p 5), in incidence of albuminuria (figure 3B, appendix p 9), or in relative change in UACR (appendix p 15). After adjustment for baseline UACR, time to albuminuria was shorter for cluster 3 (SIRD) versus cluster 2 (SIDD) in ADOPT, but not RECORD (table 1, appendix p 10). The clusters had no prediction and discrimination ability (ADOPT R^2^=0·00, C-statistic 0·52; RECORD R^2^=0·00, C-statistic 0·52); baseline UACR was a more useful measure (ADOPT R^2^=0·12, C-statistic 0·74; RECORD R^2^=0·10, C-statistic 0·73).

**Figure 4:**
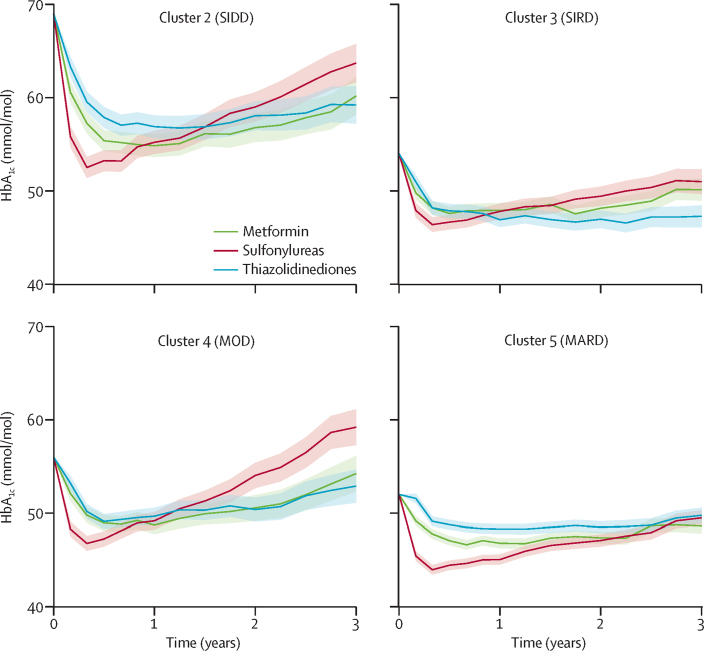
Change in HbA_1c_ by drug for clusters 2–5 in ADOPT over 3 years (n=3607) Adjusted mean HbA_1c_ over 3 years by drug. Shading shows 95% CIs. Data for cluster 1 (SAID; n=158) are shown in the appendix (p 16). SAID= severe autoimmune diabetes. SIDD=severe insulin-deficient diabetes. SIRD=severe insulin-resistant diabetes. MOD=mild obesity-related diabetes. MARD=mild age-related diabetes.

Patterns of HbA_1c_ response to the different drugs differed across clusters in ADOPT (figure 4, appendix p 16). As defined by an HbA_1c_ of greater than 3 mmol/mol compared with the other drugs, there was an overall HbA_1c_ benefit with thiazolidinedione therapy in cluster 3 (SIRD), and for sulfonylurea therapy in cluster 5 (MARD; table 2). However, the combined clinical features explained more variation in response than did the clusters: R^2^ was lower for the clusters strategy than for the clinical features strategy (In ADOPT, R^2^ 0·15 for metformin, 0·20 sulfonylureas, 0·17 thiazolidinediones for clusters strategy; R^2^ 0·35 metformin, 0·33 sulfonylureas, 0·32 thiazolidinediones for clinical features strategy).

**Table 2:** Cumulative HbA_1c_ reduction at 3 years by drug for each cluster in ADOPT

	**Cluster 1 (SAID), n=158**	**Cluster 2 (SIDD), n=759**	**Cluster 3 (SIRD), n=775**	**Cluster 4 (MOD), n=811**	**Cluster 5 (MARD), n=1262**
Metformin	−15·6 (−23·7 to −7·4)*	−35·7 (−39·9 to −31·6)*	−16·1 (−18·7 to −13·5)	−15·9 (−19·4 to −12·3)*	−12·8 (−14·5 to −11·0)
Sulfonylurea	−7·8 (−18·4 to 2·7)	−33·3 (−37·6 to −29·1)*	−15·3 (−18·1 to −12·5)	−11·2 (−14·7 to −7·7)	−16·1 (−17·9 to −14·3)*
Thiazolidinediones	−9·6 (−19·5 to 0·3)	−31·6 (−35·8 to −27·5)	−19·2 (−21·8 to −16·6)*	−15·3 (−18·6 to −12·0)*	−9·1 (−10·9 to −7·4)

Estimated as HbA_1c_ area under the curve (AUC [mmol/mol]) at 3 years (95% CI) from repeated measures mixed models. SAID=severe autoimmune diabetes. SIDD=severe insulin-deficient diabetes. SIRD=severe insulin-resistant diabetes. MOD=mild obesity-related diabetes. MARD=mild age-related diabetes.

* Individuals within the cluster randomised to drug are classified as concordant under the clusters strategy treatment selection rule: best drug for the cluster or 3-year AUC HbA_1c_ difference ≤3mmol/mol compared with best drug for the cluster.

In the independent validation cohort (RECORD) we found clinical features outperformed the clusters for treatment selection. In RECORD, we tested the performance for treatment selection of the two strategies developed in ADOPT (coefficients for the ADOPT clinical features model are given in the appendix [p 16]). Each individual in each trial was assigned as concordant or discordant with the treatment selection rule under both strategies (table 2, appendix pp 17–18).

**Figure 5:**
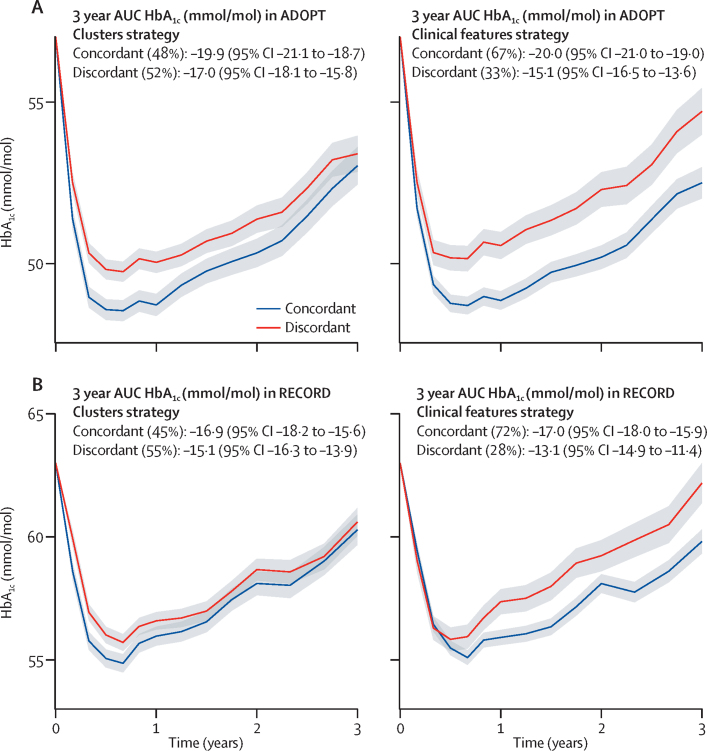
Change in HbA_1c_ over 3 years in concordant and discordant treatment selection groups (A) ADOPT development cohort (n=3785), clusters strategy (left panel) and clinical features strategy (right panel). (B) RECORD validation cohort (n=4057), clusters strategy (left panel) and clinical features strategy (right panel).

In ADOPT, using both strategies, there was a greater overall HbA_1c_ reduction in the concordant group compared with the discordant group (figure 5A). In RECORD, there was a greater benefit in the concordant group with the clinical features strategy than with the clusters strategy (figure 5B). The clinical features strategy outperformed the clusters at all HbA_1c_ thresholds that were assessed to define concordant and discordant groups in RECORD (appendix p 19).

There was no evidence of differences between clusters in the risk of cardiovascular hospitalisation or death in RECORD after adjustment for age (appendix p 20). Clusters assigned to individuals in ADOPT using the cluster-centre coordinates in ANDIS were broadly similar to those defined de-novo in ADOPT (figure 1, appendix p 21). 58% of individuals were assigned to the same cluster using the ANDIS clusters and the ADOPT clusters (appendix p 21). Differences in outcomes by ANDIS cluster are shown in the appendix (pp 22–23). ADOPT clusters outperformed the ANDIS clusters for treatment response; model performance measures were similar for glycaemic and renal progression (appendix p 24).

## Discussion

We found that the data-driven clusters of Ahlqvist and colleagues were reproducible in trial data. Clusters differed in glycaemic and renal progression but simple clinical features worked as well or better to predict disease progression (age at diagnosis for glycaemic progression and baseline renal function for renal progression). To our knowledge, for the first time we have shown differences by cluster in treatment response. However, clusters were markedly outperformed by models that used simple clinical features for the prediction of glucose-lowering response and for treatment selection. Overall, the results suggest that there will be greater clinical utility from modelling clinical features directly, rather than from using clinical features to place patients into subgroups (appendix p 25).

Although there were restricted eligibility criteria for the ADOPT and RECORD trials, subgroups defined by cluster analysis were similar to those seen in non-selective Scandinavian cohorts, and subsequently Chinese and US cohorts.^4^, ^21^ This similarity suggests that if the cluster analysis is repeated in the specified way in new datasets it will routinely produce similar clusters.

A key strength of trial data over previous observational data is the availability of protocol-driven follow-up, meaning that we were able to do a systematic assessment and show that the clusters differ in disease progression. This possibility is a considerable advantage over routine follow-up, in which therapy introduction is not protocol driven.^4^ Independently of therapy, clusters 1 (SAID), 2 (SIDD), and 4 (MOD) had an increased rate of glycaemic progression. Differences in the development of renal failure had previously been shown in observational follow-up, and we replicated a faster progression of renal disease in clusters 3 (SIRD) and 5 (MARD), although there was no evidence of a difference in renal progression after accounting for baseline renal function.

We established that the clusters differ in response to different glucose-lowering therapies. This finding was possible because of the randomised, systematic therapy given. We found a particular benefit for cluster 3 (SIRD) with thiazolidinediones, and for cluster 5 (MARD) with sulfonylureas.

The fact that clusters are reproducible and can help to predict progression and response to therapy is important. However, a key question raised in response to the original article is whether it is more clinically useful to use clinical features to assign a patient to a subgroup and then treat in a way that is best for that subgroup, or to use clinical features to predict patient outcomes directly using outcome-specific models.^7^ We found that simple clinical features were similar to or better than the clusters to stratify disease progression and to personalise therapy. A simple model incorporating only age at diagnosis was able to predict glycaemic progression as well as the clusters, having been identified as a key predictor of progression in an observational analysis.^22^ Similarly, baseline renal function explained differences between the clusters in risk of renal progression.

For treatment response we found that models combining four simple clinical measures (age, sex, baseline HbA_1c_, and BMI) explained more variation in response than did the clusters. However, this finding gives little insight into whether a strategy based on the clusters model or on a continuous features model is more useful to select between treatment options for an individual patient.^18^, ^19^ A more useful test in this context is to compare the population-level effect of applying each strategy to select treatment on glycaemic response,^19^ which we were able to directly assess, by comparing the two strategies developed in ADOPT in an independent trial dataset (RECORD). This comparison was possible because some participants in RECORD were randomly assigned to the drug estimated to be best for them on the basis of the ADOPT models (concordant group), and the remainder were randomly assigned to a drug that was not best (discordant group). The difference in HbA_1c_ between the two groups provided a measure of the population-level effect of each treatment selection strategy. In RECORD, we found a small benefit (1·8 mmol/mol over 3 years) of selecting therapy by cluster; by contrast, there was a greater benefit (3·9 mmol/mol) selecting treatment using the clinical features model (difference in 3-year HbA_1c_; figure 5B). These results suggest that attempts to personalise treatment in type 2 diabetes will have the most clinical utility if they are based on the use of continuous phenotypic measures, rather than on subgroup assignment.

Strengths of this study include the use of data from two large, long-term, randomised trials, in which we were able to not only reproduce the clustering approach of Ahlqvist and colleagues, but also to describe diabetes progression and treatment response in protocol-driven follow-up. Furthermore, we were able to test treatment selection based on clusters compared with clinical features in an independent validation dataset. The treatment selection rule we applied was designed to test clinical utility in this study, rather than to maximise outcomes for the population or individuals. Approaches to assess treatment selection strategies are not well developed and are the subject of ongoing methodological research.^18^ A limitation of our study is the potential non-representativeness of participants due to the original trial exclusion criteria. Both ADOPT and RECORD had exclusion criteria based on blood glucose levels and age (and BMI in RECORD); these clinical variables informed the cluster analysis. Despite these criteria, we found that the clusters were reproducible, with a pattern of differences in phenotypic measures that closely matched those previously reported. Given the variables informing the cluster analysis are not independent and are likely to be similarly correlated in most patients with diabetes, this reproducibility is not surprising,^7^ although similarly to the original study, we had little data on non-white ethnicities (ADOPT was 88% white, RECORD was 99%). Because of the design of the trials we were unable to assess some outcomes explored in the original study, such as time to insulin, and we did not have power to assess other outcomes, including development of end-stage renal disease. A further limitation was the therapy used in the trials; assessment of heterogeneity in treatment response for the drug classes dipeptidyl peptidase inhibitors, sodium-glucose co-transporter-2 inhibitors, and glucagon-like peptide-1 receptor agonists would be of considerable interest.

An important difference between this study and the study by Ahlqvist and colleagues is in the analysis of renal progression. Although we excluded individuals with pre-existing kidney disease, in the Scandinavian population-based cohorts people with pre-existing kidney disease when diagnosed with diabetes were not excluded and the onset of renal dysfunction was set to the first time that an abnormal value was found in clinical testing after diagnosis.

Precision medicine is successfully established in monogenic and neonatal diabetes, in which defining discrete aetiological subtypes with differing genetic causes that have different optimal treatment requirements has been possible.^23^, ^24^, ^25^ A key difference from type 2 diabetes is that the monogenic and neonatal diabetes subgroups identified have discrete and non-overlapping aetiologies and can be robustly defined by genetic sequencing. By contrast, the study by Ahlqvist and colleagues and other attempts to characterise the heterogeneity in type 2 diabetes have identified clusters with poor clinical utility because the clusters are non-aetiological, overlapping, highly dependent on the variables used to classify them, and cannot be robustly defined at an individual level.^4^, ^26^ Even genetic susceptibility clusters, which do have the advantage of being fixed throughout life, have not led to the identification of discrete aetiological subtypes of type 2 diabetes, although such clusters offer insight into mechanistic pathways underlying heterogeneity.^27^

The known heterogeneity in type 2 diabetes, together with the differences we have observed in clinical outcomes, raises the possibility of a practical clinical application of precision medicine in type 2 diabetes in the near future. Our study supports the suggestion that the optimal approach to tailor management on the basis of risk of progression and therapeutic response will be to use phenotypic measures to predict specific outcomes for individuals using multivariable models, rather than define subgroups and assume all individuals are homogeneous within each subgroup.^7^ In particular, specific clinical characteristics have been shown to have robust associations with response to specific type 2 diabetes drug options.^6^, ^28^, ^29^, ^30^ These studies raise the possibility that the relative glucose-lowering benefit of the different drugs might be identifiable by combining simple clinical measures in a model for treatment selection. Testing this possibility will require systematic assessment of associations between other patient features (including lifestyle factors, biomarkers, and concomitant medications) beyond those assessed in this study. The advantage of such an approach is that the clinical features used are already part of routine clinical care. Similarly, further systematic assessment of associations between clinical patient features and glycaemic and renal progression will be required to see whether individuals at high or low risk of progression can be robustly identified.

The methodology we have applied in this study, harnessing existing trial data at an individual level to test a precision medicine strategy developed in other data, offers an exciting, low-cost framework to assess novel precision medicine approaches without a prospective trial. Such trial datasets are increasingly available to researchers to answer secondary research questions.^31^ The approach we used of a direct comparison of different approaches in an independent dataset is a good model for defining the relative performance of such approaches. When defining the utility of models in future studies it will be important to examine multiple relevant outcomes as well as glycaemia, including cardiovascular outcomes, microvascular complications, and non-glycaemic effects of specific drugs, including weight change and side-effects.

In conclusion, we have shown that cluster-defined subgroups are reproducible and can help to define individuals that vary in the risk of diabetes progression and in glycaemic response to common therapeutic options. Our study shows that a prediction model approach combining phenotypic measures to predict specific outcomes for individual patients is likely to have greater clinical utility than is subgroup assignment. Existing trial data offer an exciting opportunity to evaluate the potential of precision medicine approaches to improve patient outcomes in type 2 diabetes.
