# Supplementary material for: Disease progression and treatment response in data-driven subgroups of type 2 diabetes compared with models based on simple clinical features: an analysis using clinical trial data
Source: Lancet Diabetes Endocrinol. 2019 Jun;7(6):442–51. doi: 10.1016/S2213-8587(19)30087-7 (PMC6520497; doi:10.1016/S2213-8587(19)30087-7)
Supplement: Supplementary appendix [file mmc1.pdf]

THE LANCET
Diabetes & Endocrinology

Supplementary appendix

This appendix formed part of the original submission and has been peer reviewed. We post it as supplied by the authors.

Supplement to: Dennis J, Shields B M, Henley W E, et al. Disease progression and treatment response in data-driven subgroups of type 2 diabetes compared with models based on simple clinical features: an analysis using clinical trial data. *Lancet Diabetes Endocrinol* 2019; published online April 29. http://dx.doi.org/10.1016/S2213-8587(19)30087-7.

Supplementary appendix for:

Disease progression and treatment response in data-driven subgroups of type 2 diabetes compared to models based on simple clinical features: an evaluation using clinical trial data

Authors: John M Dennis, Beverley M Shields, William E Henley, Angus G Jones, Andrew T Hattersley

Contents

Cluster assignment and characteristics (ADOPT and RECORD) 3
Glycaemic progression 7
Renal progression 9
HbA1c response 16
Treatment selection 17
Cardiovascular outcomes (RECORD trial) 20
Application of clusters from the Swedish All New Diabetics in Scania cohort (ANDIS) to ADOPT 21

Cluster assignment and characteristics (ADOPT and RECORD)

Supplementary Table 1: ADOPT cluster distributions, overall and by sex (n=4,003). SAID=severe autoimmune diabetes. SIDD=severe insulin-deficient diabetes. SIRD=severe insulin-resistant diabetes. MOD=mild obesity-related diabetes. MARD=mild age-related diabetes.

	Male		Female		Overall	
**Cluster**	**N**	**%**	**N**	**%**	**N**	**%**
1 SAID	94	4%	74	4%	168	4%
2 SIDD	506	22%	302	18%	808	20%
3 SIRD	448	19%	369	22%	817	20%
4 MOD	411	18%	447	26%	858	21%
5 MARD	844	37%	508	30%	1352	34%

Supplementary Table 2: Cluster centre coordinates in ADOPT

	Cluster	HbA1c	BMI	Age at diagnosis	HOMA2-B	HOMA2-IR
Females	C2 (SIDD)	1.357582	-0.438702	0.209430	-0.873420	-0.508708
	C3 (SIRD)	-0.207560	0.801772	-0.048181	1.168571	1.276217
	C4 (MOD)	-0.283972	0.282755	-0.956176	-0.257172	-0.274304
	C5 (MARD)	-0.406427	-0.570389	0.751853	-0.103295	-0.383230
Males	C2 SIDD)	1.146754	-0.334983	-0.300259	-0.780702	-0.448964
	C3 (SIRD)	-0.419911	0.021167	0.587122	1.132740	0.960985
	C4 (MOD)	0.102709	1.357982	-0.838457	0.480047	0.743829
	C5 (MARD)	-0.514633	-0.471697	0.276666	-0.366980	-0.603150

Supplementary Table 3: ADOPT Cluster characteristics by sex (n=4,003). SAID=severe autoimmune diabetes. SIDD=severe insulin-deficient diabetes. SIRD=severe insulin-resistant diabetes. MOD=mild obesity-related diabetes. MARD=mild age-related diabetes. HOMA2-B=homoeostatic model assessment 2 estimates of β-cell function. HOMA2-IR=homoeostatic model assessment 2 estimates of insulin resistance.

A) Females

	Number of participants (%)	HbA1c (mmol/mol)		BMI kg/m2		Age at diagnosis (years)		HOMA2-B (%)		HOMA2-IR (%)	
Cluster		Median	IQR	Median	IQR	Median	IQR	Median	IQR	Median	IQR
1 SAID	74 (4%)	59	50-65	33	28-38	59	51-64	63	46-87	2.4	1.5-3.4
2 SIDD	302 (18%)	69	65-75	30	27-34	57	52-64	49	38-59	2.2	1.7-2.8
3 SIRD	369 (22%)	55	49-61	39	35-43	55	49-62	102	87-125	4.3	3.8-5.0
4 MOD	447 (26%)	54	50-58	35	31-40	46	41-50	67	54-79	2.6	2.1-3.1
5 MARD	508 (30%)	53	49-57	30	27-33	64	58-68	70	58-83	2.5	1.9-3.0

B) Males

	Number of participants (%)	HbA1c (mmol/mol)		BMI kg/m2		Age at diagnosis (years)		HOMA2-B (%)		HOMA2-IR (%)	
Cluster		Median	IQR	Median	IQR	Median	IQR	Median	IQR	Median	IQR
1 SAID	94 (4%)	57	53-64	29	27-33	57	49-64	60	46-77	2.4	1.7-3.1
2 SIDD	506 (22%)	67	63-73	29	27-32	53	46-60	49	38-59	2.3	1.8-2.8
3 SIRD	448 (19%)	52	48-57	31	29-34	63	57-68	100	86-117	3.7	3.2-4.6
4 MOD	411 (18%)	57	52-63	37	34-42	49	41-54	83	68-98	3.5	3.0-4.3
5 MARD	844 (37%)	52	48-56	28	26-31	59	53-65	60	51-72	2.2	1.7-2.6

Supplementary Table 4: Cluster characteristics for each trial population

ADOPT (n=4,003). Median (interquartile range) unless stated.

	1 SAID	2 SIDD	3 SIRD	4 MOD	5 MARD
N. participants (%)	168 (4%)	808 (20%)	817 (20%)	858 (21%)	1352 (34%)
HbA1c (mmol/mol)	58 (52-64)	67 (64-74)	53 (48-60)	55 (51-61)	53 (49-56)
BMI (kg/m²)	30 (27-36)	29 (27-32)	34 (30-38)	36 (33-40)	29 (26-31)
Age at diagnosis (years)	57 (49-64)	55 (48-61)	59 (53-66)	47 (41-52)	61 (55-66)
HOMA2-B (%)*	61 (46-83)	49 (38-59)	101 (87-121)	74 (59-89)	64 (53-76)
HOMA2-IR*	2.4 (1.6-3.3)	2.3 (1.8-2.8)	4.0 (3.4-4.7)	3.1 (2.4-3.7)	2.3 (1.8-2.7)
Male sex (%)	94 (56%)	506 (63%)	448 (55%)	411 (48%)	844 (62%)
Ethnicity (% White)	158 (94%)	745 (92%)	804 (98%)	801 (93%)	1327 (98%)
Fasting glucose (mmol/l)	8.3 (7.6-9.3)	9.2 (8.4-10.2)	7.9 (7.2-8.7)	8.3 (7.5-9.2)	8.0 (7.4-8.6)
Fasting insulin (pmol/L)	108 (70-150)	93 (72-129)	208 (150-280)	158 (114-215)	96 (72-126)
Fasting C-peptide (nmol/L)	0.9 (0.6-1.3)	0.8 (0.7-1.0)	1.6 (1.4-1.8)	1.2 (1.0-1.4)	0.9 (0.7-1.1)
eGFR (ml/min per 1.73m²)**	93 (82-103)	98 (87-106)	90 (77-100)	104 (96-112)	93 (82-100)
eGFR <60 at baseline (%)**	4 (2%)	14 (2%)	41 (5%)	8 (1%)	44 (3%)
Albuminuria (mg/g)***	7 (4-16)	8 (4-17)	8 (4-18)	7 (4-19)	6 (4-13)
Albuminuria ≥ 30 at baseline (%)***	26 (16%)	126 (16%)	145 (18%)	154 (18%)	158 (12%)
HDL (mmol/L)	1.2 (1.1-1.5)	1.2 (1.0-1.5)	1.1 (1.0-1.3)	1.1 (1.0-1.4)	1.3 (1.1-1.5)
LDL (mmol/L)	3.0 (2.4-3.6)	3.3 (2.7-4.0	2.9 (2.4-3.6)	3.1 (2.5-3.7)	3.2 (2.6-3.8)
ALT (U/L)	21 (16-31)	22 (17-31)	26 (19-36)	26 (18-37)	21 (16-29)

*Calculated with HOMA2 calculator using fasting glucose and fasting C-peptide
** Calculated with CKD-EPI formula ***71 individuals with missing albuminuria at baseline

RECORD (n=4,148; ADOPT-defined clusters). Median (interquartile range) unless stated.

	1 SAID	2 SIDD	3 SIRD	4 MOD	5 MARD
N. participants (%)	NA	974 (23%)	803 (19%)	852 (21%)	1519 (37%)
HbA1c (mmol/mol)		72 (68-75)	58 (55-64)	62 (57-66)	60 (55-63)
BMI (kg/m²)		29 (27-32)	34 (31-37)	35 (31-37)	29 (27-31)
Age at diagnosis (years)		50 (44-55)	54 (48-59)	44 (40-48)	56 (51-61)
HOMA2-B (%)*		18 (13-24)	57 (45-74)	32 (23-42)	28 (20-36)
HOMA2-IR*		1.1 (0.7-1.5)	2.4 (1.9-3.1)	1.4 (1.0-2.0)	1.0 (0.7-1.3)
Diabetes duration (years)		7 (4-11)	5 (3-7)	6 (4-10)	5 (3-8)
Male sex (%)		571 (59%)	361 (45%)	313 (37%)	898 (59%)
Ethnicity (% White)		964 (99%)	795 (99%)	841 (99%)	1510 (99%)
Fasting glucose (mmol/l)		11 (10-13)	9 (8-10)	10 (8-11)	9 (8-10)
Fasting insulin (pmol/L)		48 (32-66)	114 (91-146)	67 (48-91)	45 (32-61)
Fasting C-peptide (nmol/L)		NA	NA	NA	NA
eGFR (ml/min per 1.73m²)**		100 (91-106)	97 (88-105)	106 (99-112)	96 (87-102)
eGFR <60 at baseline (%)**		13 (1%)	28 (3%)	9 (1%)	30 (2%)
Albuminuria (mg/g)***		9 (5-25)	9 (5-23)	9 (5-24)	8 (4-17)
Albuminuria ≥ 30 at baseline (%)***		190 (22%)	142 (20%)	149 (20%)	209 (16%)
HDL (mmol/L)		1.2 (1.0-1.4)	1.1 (0.9-1.3)	1.2 (1.0-1.4)	1.2 (1.0-1.4)
LDL (mmol/L)		3.4 (2.8-4.0)	3.2 (2.5-3.8)	3.2 (2.6-3.8)	3.3 (2.6-3.8)
ALT (U/L)		25 (19-36)	29 (21-41)	26 (19-39)	23 (17-31)

*Calculated with HOMA2 calculator using fasting glucose and fasting insulin as fasting C-peptide not available
** Calculated with CKD-EPI formula, 2 individuals missing eGFR at baseline ***479 individuals with missing albuminuria at baseline

Supplementary Figure 1: clusters characteristics in RECORD. Cluster distribution and cluster characteristics (n=4,148). RECORD participants assignment and distributions of baseline clinical characteristics according to k-means clustering (A) Clusters derived in ADOPT and assigned to RECORD participants (B) Clusters derived in RECORD and assigned to RECORD participants.

SAID=severe autoimmune diabetes. SIDD=severe insulin-deficient diabetes. SIRD=severe insulin-resistant diabetes. MOD=mild obesity-related diabetes. MARD=mild age-related diabetes. HOMA2-B=homoeostatic model assessment 2 estimates of β-cell function. HOMA2-IR=homoeostatic model assessment 2 estimates of insulin resistance.

(A) ADOPT derived clusters

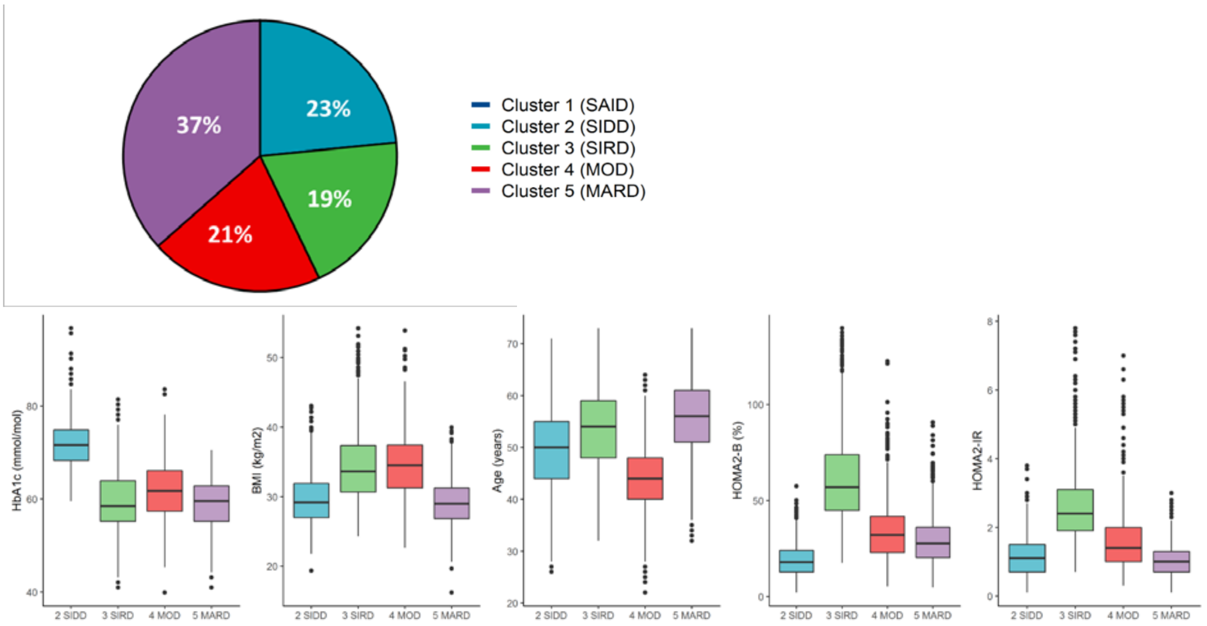

(B) RECORD derived clusters

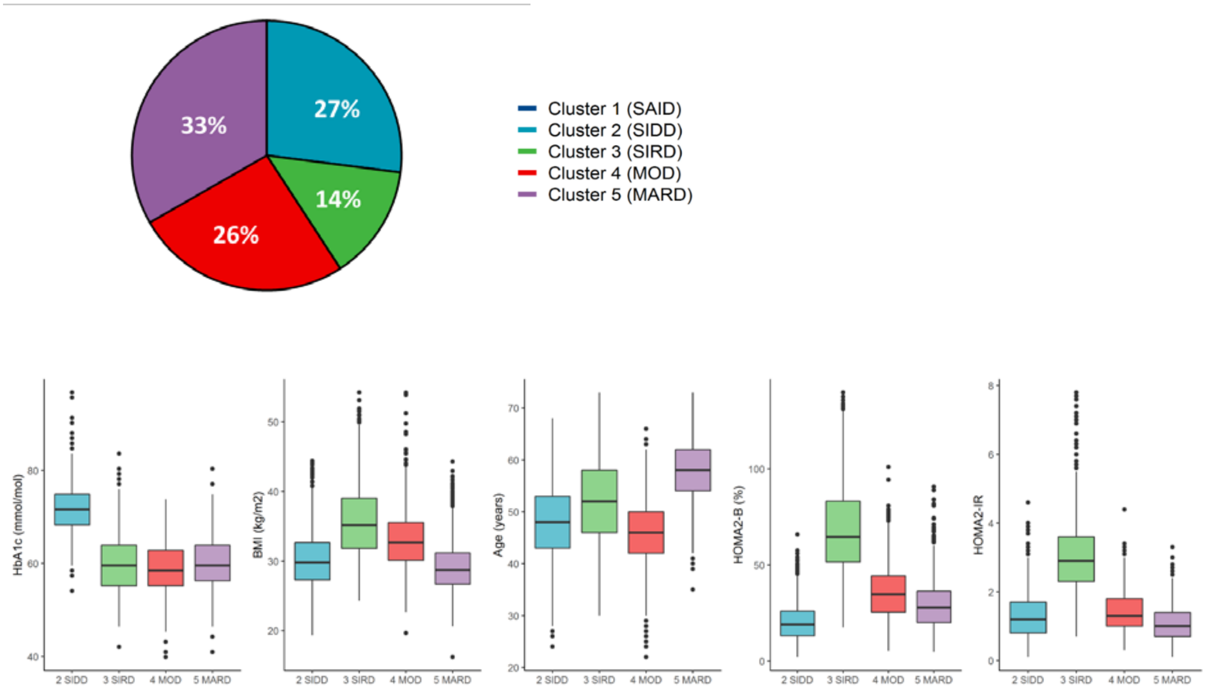

Glycaemic progression

Supplementary Figure 2: HbA1c over time from randomisation by cluster in ADOPT (n=3,802).

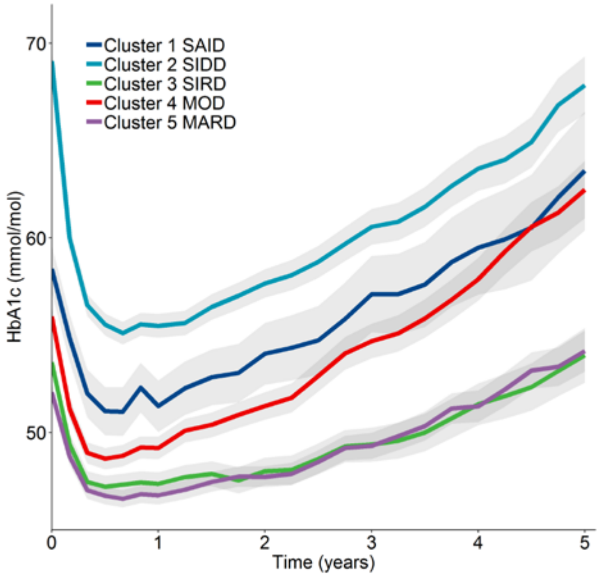

Supplementary Figure 3: HbA1c in RECORD

(A) HbA1c over time from randomisation by cluster (n=4,057);

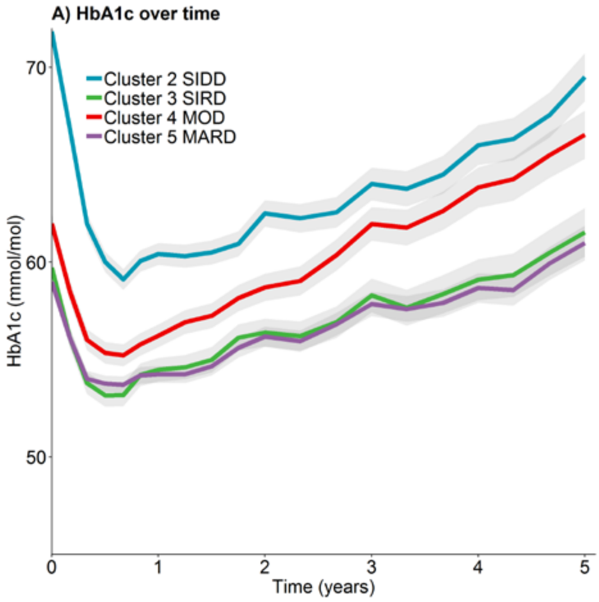

Supplementary Figure 3 (cont.): HbA1c in RECORD.

(B) Glycaemic progression from 1 year by ADOPT derived cluster (n=3,586); (C) Glycaemic progression from 1 year by age at diagnosis (10th, 50th and 90% percentile of RECORD participants) (n=3,586). Data are estimates from repeated measures mixed effects models.

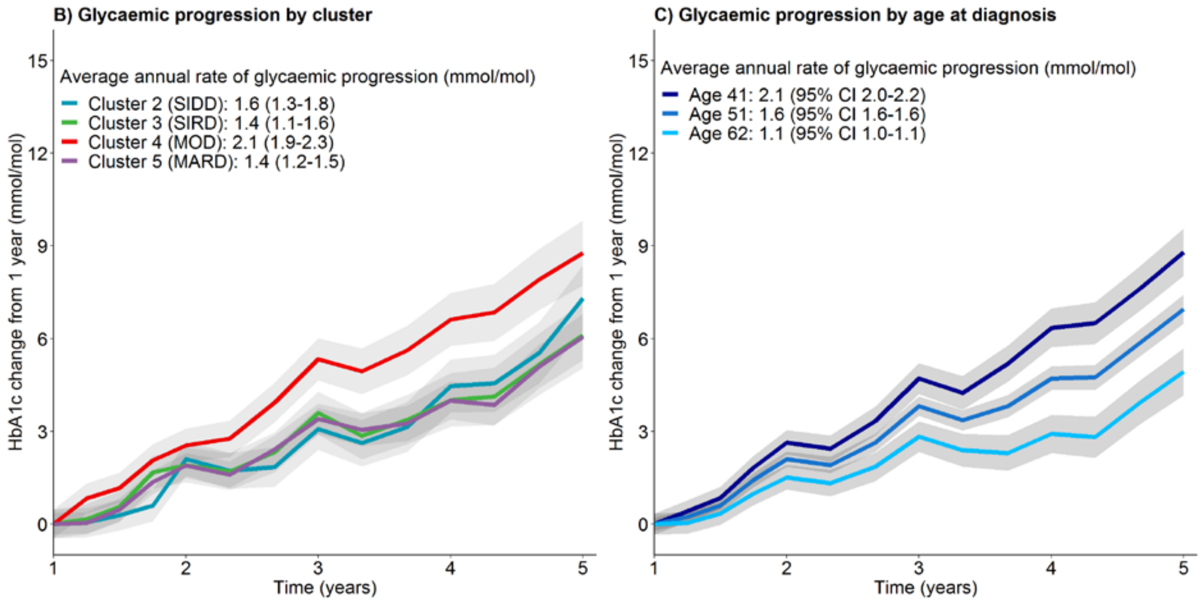

Supplementary Table 5: Glycaemic progression model performance measures to compare model using clusters and model using age at diagnosis. A higher adequacy index suggests a better model (calculated as model LR x^2 / Combined model LR x^2)

A) ADOPT

	R^2	AIC	LR x^2	Adequacy Index
Clusters	0.084	221404	1225	0.95
Age at diagnosis	0.088	221318	1210	0.94
Combined model (clusters + age at diagnosis)	0.093	221371	1292	1.00

B) RECORD

	R^2	AIC	LR x^2	Adequacy Index
Clusters	0.048	274658	1065	0.89
Age at diagnosis	0.052	274624	1099	0.92
Combined model (clusters + age at diagnosis)	0.055	274642	1196	1.00

Renal progression

Supplementary Figure 4: Renal progression by cluster in RECORD (clusters derived from ADOPT)

(A) Cumulative incidence of CKD Stage 3 (confirmed eGFR <60) in individuals with eGFR ≥60 at baseline (n=4,066). eGFR calculated using CKD-EPI formula.

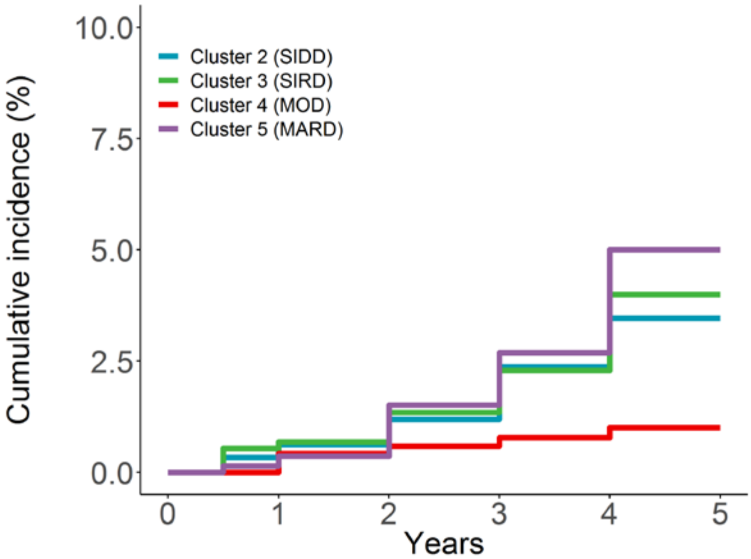

Number at risk

	0	1	2	3	4	5
Cluster 2 (SIDD)	961	699	524	419	358	281
Cluster 4 (MOD)	842	713	591	517	441	374
Cluster 5 (MARD)	1488	1335	1214	1092	966	826

(B) Cumulative incidence of albuminuria (UACR ≥30 mg/g) in individuals with UACR <30 mg/g at baseline (n=2,979).

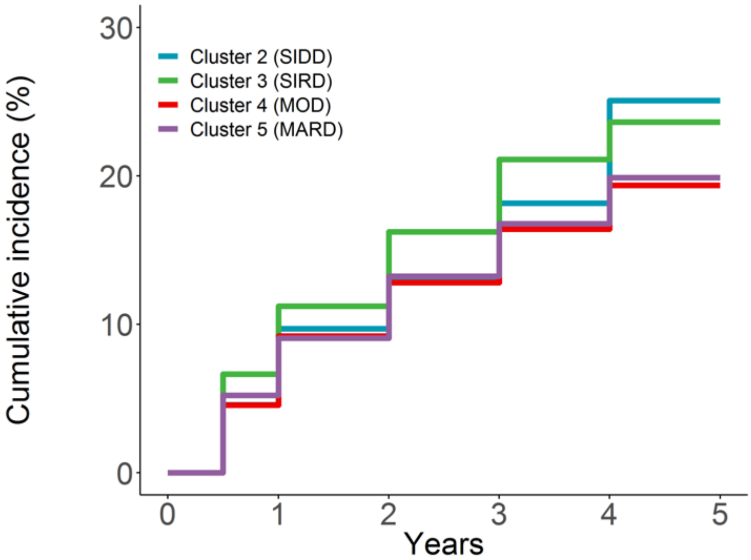

Number at risk

	0	1	2	3	4	5
Cluster 2 (SIDD)	680	486	344	275	225	167
Cluster 3 (SIRD)	580	490	408	343	283	233
Cluster 4 (MOD)	605	493	403	339	283	231
Cluster 5 (MARD)	1114	962	848	740	643	546

Supplementary Table 6: Risk of renal progression by cluster in RECORD (clusters derived from ADOPT)

(A) Time to CKD Stage 3 (n=4,066). eGFR calculated using CKD-EPI formula.

	**No.**	**Person years at risk**	**Events**	**Hazard ratio (95% CI)**	
				**Unadjusted**	**Adjusted***
**Time to CKD**					
Cluster					
C1 (SAID)	NA	NA	NA	NA	NA
C2 (SIDD)	961	2551	17	1.00 (ref)	1.00 (ref)
C3 (SIRD)	775	2789	22	1.12 (0.60-2.11)	0.96 (0.51-1.81)
C4 (MOD)	842	2811	6	0.31 (0.12-0.78)	0.57 (0.22-1.45)
C5 (MARD)	1488	5658	55	1.37 (0.79-2.36)	1.16 (0.67-2.00)

*Adjusted for baseline eGFR

(B) Time to albuminuria (n=2,979)

	**No.**	**Person years at risk**	**Events**	**Hazard ratio (95% CI)**	
				**Unadjusted**	**Adjusted***
**Time to albuminuria**					
Cluster					
C1 (SAID)	NA	NA	NA	NA	NA
C2 (SIDD)	680	1679	103	1.00 (ref)	1.00 (ref)
C3 (SIRD)	580	1860	113	1.04 (0.80-1.36)	1.02 (0.78-1.34)
C4 (MOD)	605	1869	90	0.82 (0.62-1.09)	0.82 (0.62-1.09)
C5 (MARD)	1114	3906	188	0.85 (0.66-1.08)	0.92 (0.72-1.17)

*Adjusted for baseline UACR

Supplementary Table 7: Time to CKD Stage 3. eGFR calculated using MDRD formula.

(A) ADOPT (n=3,650)

	**No.**	**Person years at risk**	**Events**	**Hazard ratio (95% CI)**	
				**Unadjusted**	**Adjusted***
**Time to CKD**					
Cluster					
C1 (SAID)	152	492	7	3.00 (1.16-7.72)	1.67 (0.64-4.32)
C2 (SIDD)	748	2235	11	1.00 (ref)	1.00 (ref)
C3 (SIRD)	729	2427	35	2.99 (1.53-5.92)	1.65 (0.84-3.26)
C4 (MOD)	799	2406	11	0.93 (0.40-2.14)	1.33 (0.57-3.06)
C5 (MARD)	1222	4325	41	2.00 (1.03-3.90)	1.52 (0.78-2.97)

*Adjusted for baseline eGFR

(B) RECORD (n=4,032)

	**No.**	**Person years at risk**	**Events**	**Hazard ratio (95% CI)**	
				**Unadjusted**	**Adjusted***
**Time to CKD**					
Cluster					
C1 (SAID)	NA	NA	NA	NA	NA
C2 (SIDD)	956	2528	20	1.00 (ref)	1.00 (ref)
C3 (SIRD)	769	2753	30	1.31 (0.74-2.31)	1.10 (0.91-1.94)
C4 (MOD)	838	2781	15	0.66 (0.34-1.28)	0.98 (0.50-1.91)
C5 (MARD)	1469	5570	74	1.58 (0.96-2.59)	1.41 (0.86-2.32)

*Adjusted for baseline eGFR

Supplementary Figure 5: Relative change in eGFR from baseline, by cluster. eGFR calculated using CKD-EPI formula. Estimates are from mixed effects models.

A) ADOPT (n=3,694)

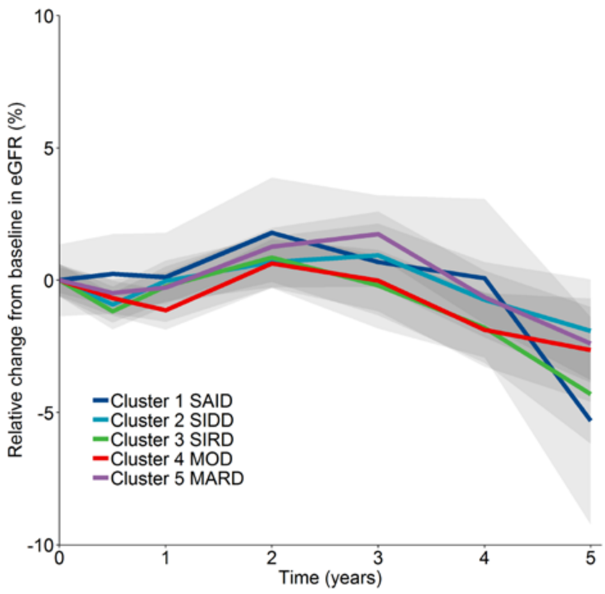

B) RECORD (n=4,066)

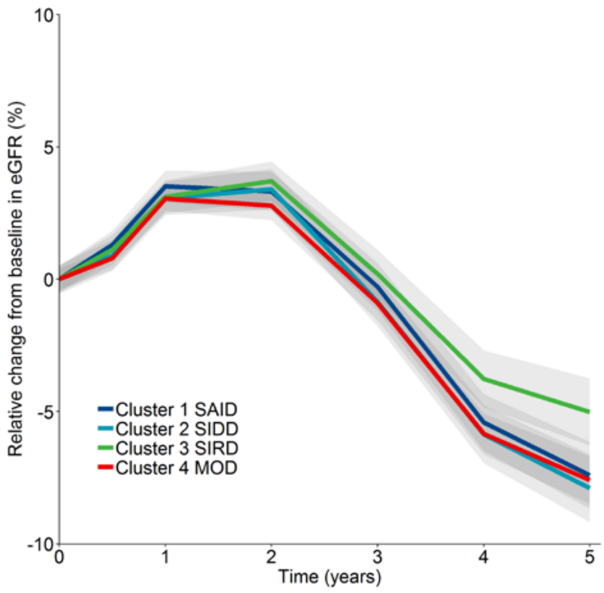

Supplementary Figure 6: Cumulative incidence of 30% relative change in eGFR from baseline, by cluster. eGFR calculated using CKD-EPI formula.

A) ADOPT (n=3,694)

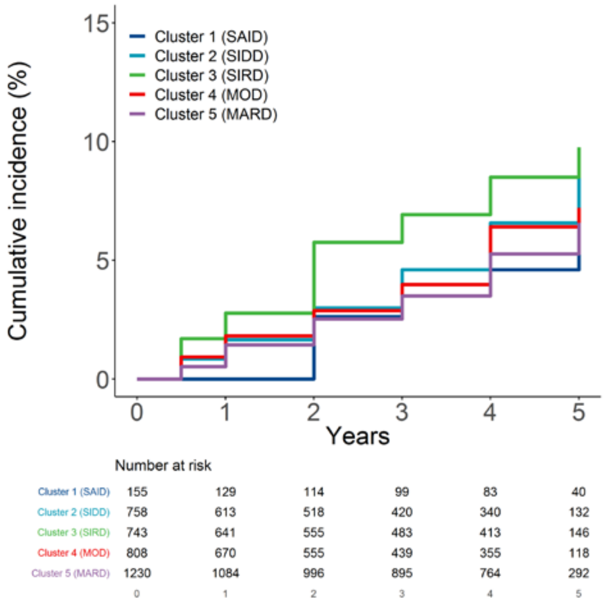

B) RECORD (n=4,066)

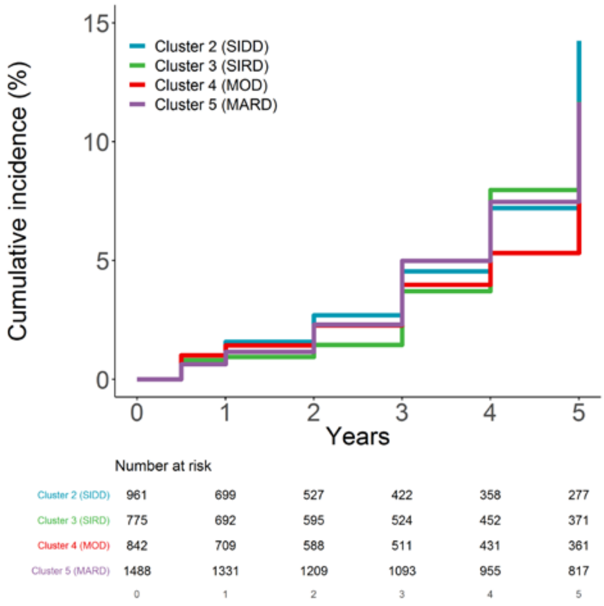

Supplementary Table 8: Risk of 30% relative change in eGFR from baseline by cluster. eGFR calculated using CKD-EPI formula.

(A) ADOPT (n=3,694)

	**No.**	**Person years at risk**	**Events**	**Hazard ratio (95% CI)**	
				**Unadjusted**	**Adjusted***
**Time to 30% relative change in eGFR**					
Cluster					
C1 (SAID)	155	508	7	0.88 (0.39-1.97)	0.78 (0.35-1.77)
C2 (SIDD)	758	2239	35	1.00 (ref)	1.00 (ref)
C3 (SIRD)	743	2452	51	1.33 (0.87-2.05)	1.16 (0.75-1.79)
C4 (MOD)	808	2387	34	0.92 (0.57-1.48)	1.05 (0.65-1.69)
C5 (MARD)	1230	4359	54	0.79 (0.51-1.20)	0.72 (0.47-1.10)

*Adjusted for baseline eGFR

(B) RECORD (n=4,066)

	**No.**	**Person years at risk**	**Events**	**Hazard ratio (95% CI)**	
				**Unadjusted**	**Adjusted***
**Time to 30% relative change in eGFR**					
Cluster					
C1 (SAID)	NA	NA	NA	NA	NA
C2 (SIDD)	961	2547	58	1.00 (ref)	1.00 (ref)
C3 (SIRD)	775	2771	57	0.83 (0.58-1.20)	0.78 (0.54-1.12)
C4 (MOD)	842	2773	40	0.59 (0.39-0.88)	0.74 (0.49-1.11)
C5 (MARD)	1488	5625	122	0.85 (0.62-1.16)	0.78 (0.57-1.07)

*Adjusted for baseline eGFR

Supplementary Figure 7: Relative change in urinary albumin to creatinine ratio from baseline, by cluster. Estimates are from mixed effects models with UACR modelled on log scale.

A) ADOPT (n=3,168)

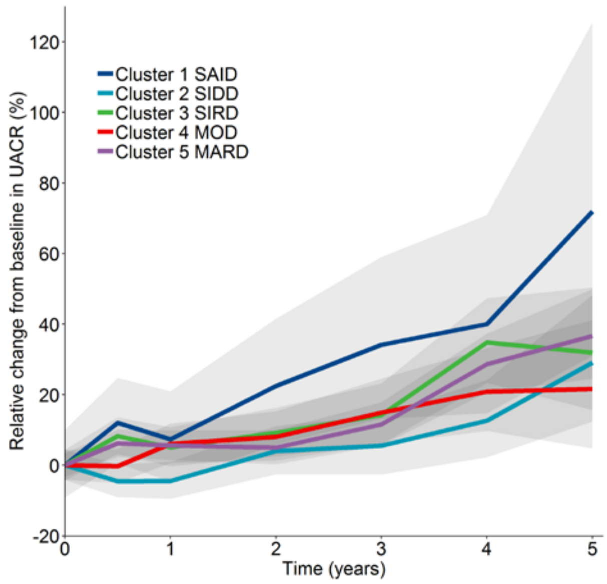

B) RECORD (n=2,979)

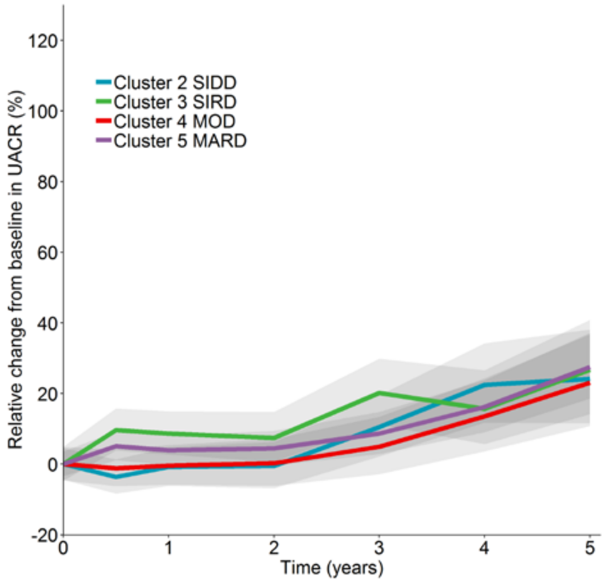

HbA1c response

Supplementary Figure 8: Changes in HbA1c (ADOPT trial, n=3,785). Adjusted mean HbA1c over 3 years by drug for clusters 1-5 (repeated measures mixed model). Grey shading shows 95% CIs.

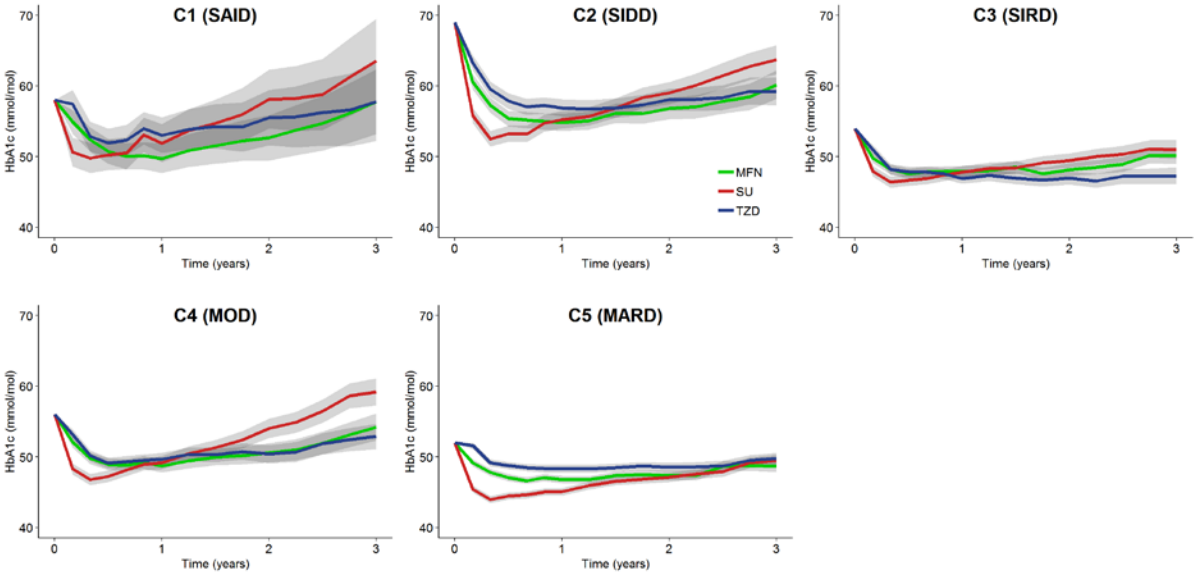

Supplementary Table 9: Beta coefficients from mixed effects models for clinical features, by drug. For continuous features beta coefficients represent the change in HbA1c response for a 1-unit increase in the clinical feature. A negative coefficient indicates a higher value of the clinical feature is associated with greater reduction in HbA1c.

	Metformin	Sulfonylureas	Thiazolidinediones
Baseline HbA1c (time 0)*	0.69 (0.66;0.72)	0.59 (0.55;0.63)	0.69 (0.65;0.73)
BMI	-0.02 (-0.07;0.03)	0.03 (-0.02;0.09)	-0.11 (-0.16;-0.06)
Age at diagnosis	0.00 (-0.03;0.03)	-0.02 (-0.05;0.01)	-0.02 (-0.06;0.01)
Sex: Male	0.53 (-0.07;1.13)	-1.54 (-2.19;-0.89)	0.59 (-0.06;1.23)

*Full baseline HbA1c:study visit interaction terms not reported for brevity.

Treatment selection

Supplementary Table 10

ADOPT number of concordant individuals, by cluster, for treatment selection at 3 years based on Strategy A) treatment selection based on clusters

	**Discordant**	**Concordant**
Cluster		
C1 (SAID)	93	65
C2 (SIDD)	257	502
C3 (SIRD)	510	265
C4 (MOD)	272	539
C5 (MARD)	838	424

Supplementary Table 11

ADOPT number (%) of concordant individuals, by drug at 3 years, for:

Strategy A) treatment selection based on clusters

	Discordant	Concordant
Overall	1970 (52%)	1795 (48%)
By randomised drug:		
Metformin	702 (55%)	569 (45%)
Sulfonylureas	555 (45%)	672 (55%)
Thiazolidinedione	713 (56%)	554 (44%)

Strategy B) treatment selection based on clinical features

	**Discordant**	**Concordant**
Overall	1227 (33%)	2538 (67%)
By randomised drug:		
Metformin	225 (18%)	1046 (82%)
Sulfonylureas	455 (37%)	772 (63%)
Thiazolidinedione	547 (43%)	720 (57%)

Supplementary Table 12

RECORD number of concordant individuals, by cluster, for treatment selection at 3 years based on Strategy A) treatment selection based on clusters

	Discordant	Concordant
Cluster		
C1 (SAID)	-	-
C2 (SIDD)	455	493
C3 (SIRD)	406	386
C4 (MOD)	239	594
C5 (MARD)	1121	363

Supplementary Table 13

RECORD number (%) of concordant individuals, by drug at 3 years, for:

Strategy A) treatment selection based on clusters

	Discordant	Concordant
Overall	2221 (55%)	1836 (45%)
By randomised drug:		
Metformin	540 (54%)	463 (46%)
Sulfonylureas	469 (46%)	546 (54%)
Thiazolidinedione	1212 (59%)	827 (41%)

Strategy B) treatment selection based on clinical features

	Discordant	Concordant
Overall	1117 (28%)	2940 (72%)
By randomised drug:		
Metformin	23 (2%)	980 (98%)
Sulfonylureas	494 (49%)	521 (51%)
Thiazolidinedione	600 (29%)	1439 (71%)

Supplementary Table 14: Change in AUC HbA1c over three years in concordant and discordant treatment selection groups using different HbA1c thresholds to define concordant/discordant groups, for clusters model and clinical features model (RECORD n=4,057)

HbA1c threshold (mmol/mol	Clusters 3 Year AUC HbA1c		Continuous features 3 Year AUC HbA1c	
	Concordant	Discordant	Concordant	Discordant
0	-18.0 (-19.6;-16.4)	-15.0 (-16.1;-14.0)	-18.3 (-20.0;-16.7)	-14.8 (-15.9;-13.8)
1	-17.0 (-18.4;-15.6)	-15.2 (-16.3;-14.0)	-18.3 (-19.6;-16.9)	-13.9 (-15.1;-12.7)
2	-17.0 (-18.4;-15.6)	-15.2 (-16.3;-14.0)	-17.6 (-18.7; -16.5)	-13.2 (-14.7;-11.8)
3	-16.9 (-18.2;-15.6)	-15.1 (-16.3;-13.9)	-17.0 (-18.0;-15.9)	-13.1 (-14.9;-11.4)
4	-16.9 (-18.1;-15.7)	-14.9 (-16.2;-13.6)	-16.6 (-17.5;-15.6)	-13.4 (-15.4;-11.4)

Cardiovascular outcomes (RECORD trial)

Supplementary Figure 9: Cumulative incidence of cardiovascular hospitalisation or death, by ADOPT-derived cluster.

RECORD (n=4,057)

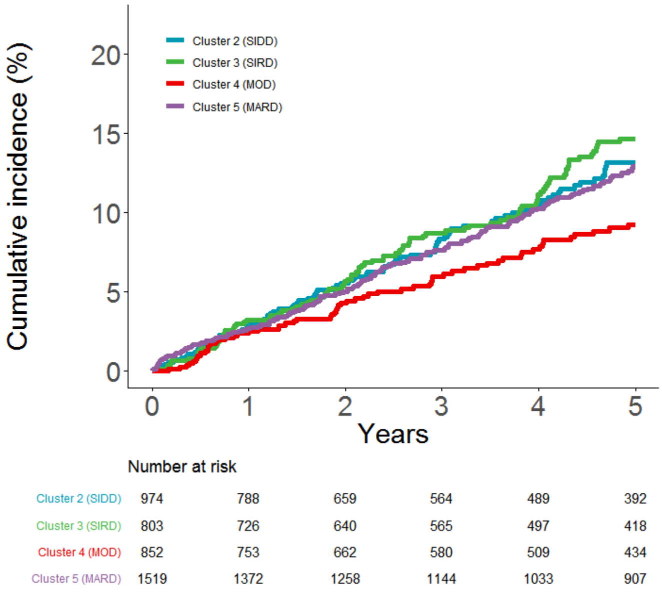

Supplementary Table 15: Risk of cardiovascular hospitalisation or death by cluster in RECORD (clusters derived from ADOPT)

RECORD (n=4,057)

	**No.**	**Person years at risk**	**Events**	**Hazard ratio (95% CI)**	
				**Unadjusted**	**Adjusted***
**Time to cardiovascular hospitalisation or death**					
Cluster					
C1 (SAID)	NA	NA	NA	NA	NA
C2 (SIDD)	948	3172	88	1.00 (ref)	1.00 (ref)
C3 (SIRD)	792	3038	94	1.11 (0.83-1.49)	1.06 (0.79-1.41)
C4 (MOD)	833	3141	62	0.71 (0.51-0.98)	1.02 (0.73-1.43)
C5 (MARD)	1484	5996	161	0.97 (0.74-1.25)	0.79 (0.61-1.03)

*Adjusted for age at trial entry

Application of clusters from the Swedish All New Diabetics in Scania cohort (ANDIS) to ADOPT

Supplementary Figure 10: Characteristics of clusters assigned in ADOPT from the cluster centre coordinates in ANDIS (n=4,003). Cluster centre coordinates originally published in Table S3, Ahlqvist et al., Lancet Diabetes Endocrinology 2018;6:361-69.

SAID=severe autoimmune diabetes. SIDD=severe insulin-deficient diabetes. SIRD=severe insulin-resistant diabetes. MOD=mild obesity-related diabetes. MARD=mild age-related diabetes. HOMA2-B=homoeostatic model assessment 2 estimates of β-cell function. HOMA2-IR=homoeostatic model assessment 2 estimates of insulin resistance.

(A) Distribution of ADOPT participants according to ANDIS clustering

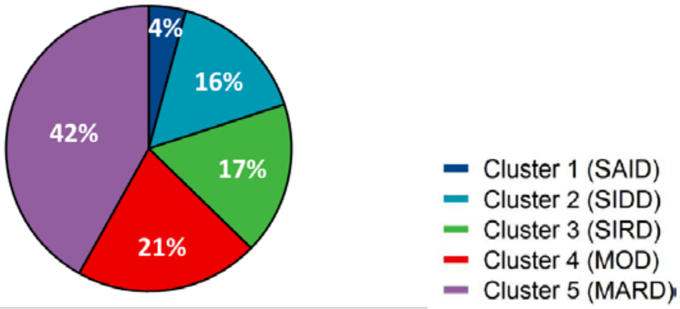

(B) Distributions of HbA1c, BMI, age at diagnosis, HOMA2-B, and HOMA2-IR at baseline for each ANDIS-derived cluster.

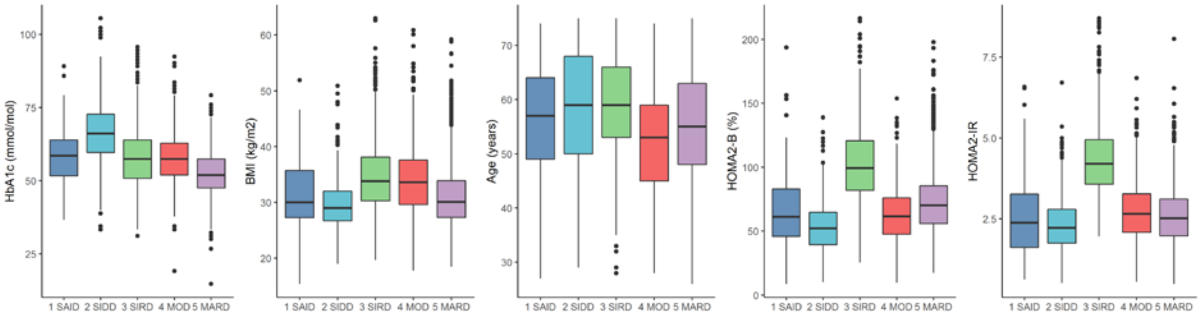

Supplementary Table 16: Concordance between clusters defined de-novo in ADOPT and clusters assigned in ADOPT from ANDIS cluster centre coordinates

	ANDIS clusters				
ADOPT clusters	C1 (SAID)	C2 (SIDD)	C3 (SIRD)	C4 (MOD)	C5 (MARD)
C1 (SAID)	**100%**	0%	0%	0%	0%
C2 (SIDD)	0%	**56%**	9%	25%	9%
C3 (SIRD)	0%	1%	**59%**	2%	38%
C4 (MOD)	0%	2%	12%	**43%**	43%
C5 (MARD)	0%	11%	3%	18%	**68%**

Supplementary Figure 11: Glycaemic progression by cluster in ADOPT from one to five years using ANDIS-derived clusters (n=3,016)

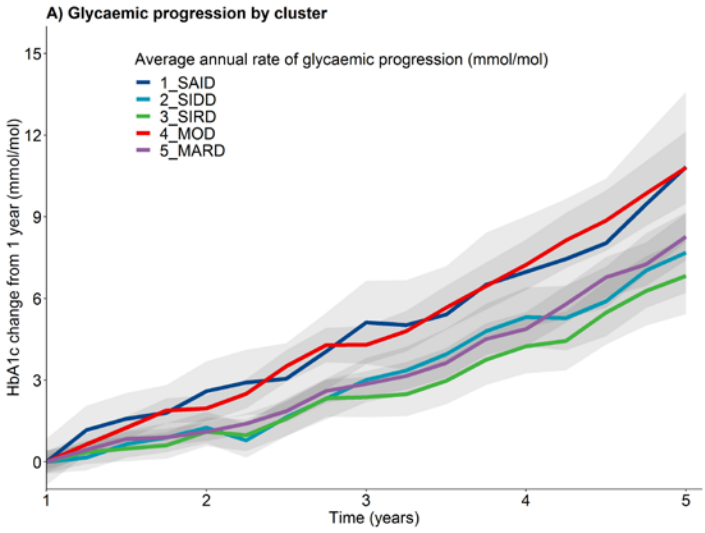

Supplementary Figure 12: Renal progression by cluster in ADOPT over five years using ANDIS-derived clusters.

(A) Cumulative incidence of CKD Stage 3 (confirmed eGFR <60) in individuals with eGFR ≥60 at baseline (n=3,694). eGFR calculated using CKD-EPI formula.

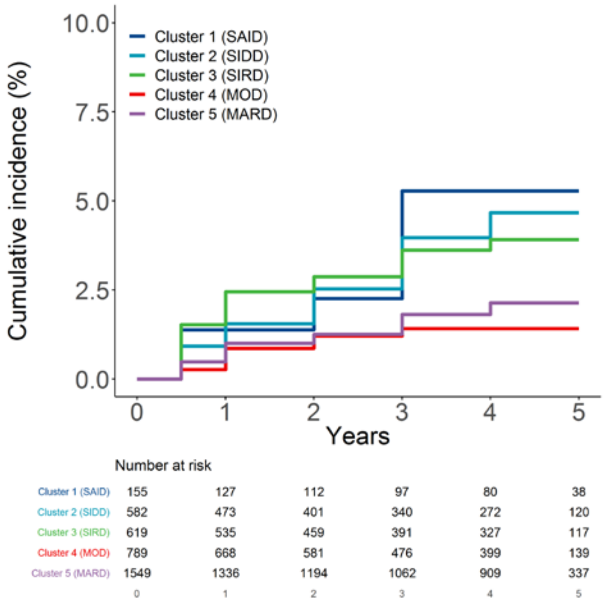

(B) Cumulative incidence of albuminuria (UACR ≥30 mg/g) in individuals with UACR <30 mg/g at baseline (n=3,168).

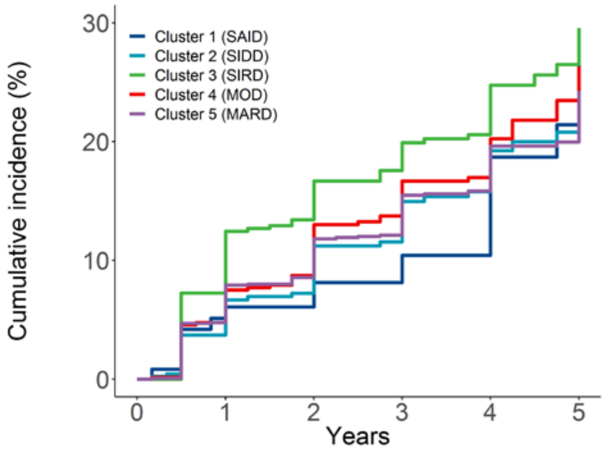

Number at risk

	0	1	2	3	4	5
Cluster 1 (SAID)	129	101	91	80	65	27
Cluster 2 (SIDD)	492	392	326	259	194	88
Cluster 3 (SIRD)	533	429	344	281	228	72
Cluster 4 (MOD)	656	522	446	351	279	81
Cluster 5 (MARD)	1358	1120	984	837	665	222

Supplementary Figure 13: Change in HbA1c by drug for each cluster in ADOPT over three years using ANDIS-derived clusters (n=3,785). Adjusted mean HbA1c over three years by drug. Grey shading shows 95% confidence intervalss.

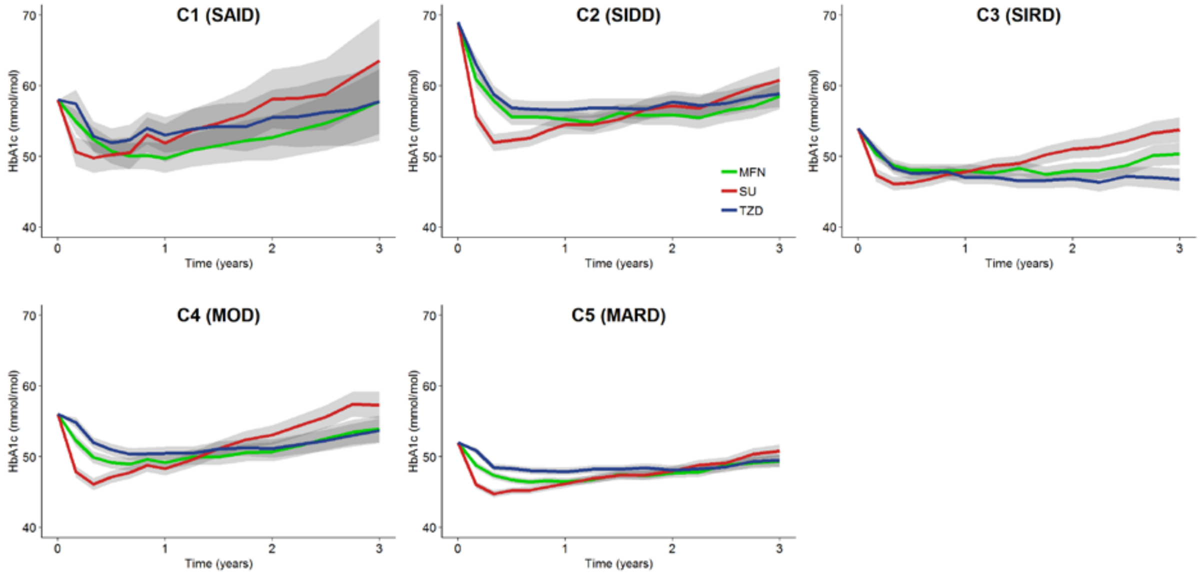

Supplementary Table 17: Model performance measures to compare clusters defined de-novo in ADOPT and clusters assigned in ADOPT from ANDIS cluster centre coordinates

A) Glycaemic progression from one to five years (n=3,016)

	R^2	AIC
ADOPT clusters	0.084	221404
ANDIS clusters	0.078	221446

B) Time to CKD Stage 3 (confirmed eGFR <60) in individuals with eGFR ≥60 at baseline (n=3,694). eGFR calculated using CKD-EPI formula.

	C-statistic	R^2
ADOPT clusters	0.58	0.01
ANDIS clusters	0.59	0.01

C) Time to albuminuria (UACR ≥30 mg/g) in individuals with UACR <30 mg/g at baseline (n=3,168).

	C-statistic	R^2
ADOPT clusters	0.52	0.002
ANDIS clusters	0.52	0.003

D) Treatment response (changes in HbA1c over 3 years) explained variation (R^2)

	Metformin	Sulfonylurea	Thiazolidinedione
ADOPT clusters	0.15	0.20	0.17
ANDIS clusters	0.10	0.12	0.09
